# Effect of unilateral intrathecal prilocaine–fentanyl versus bupivacaine–fentanyl on postoperative spontaneous voiding in ambulatory male patients undergoing inguinal hernioplasty: a randomized double-blinded comparative study

**DOI:** 10.1038/s41598-026-59157-8

**Published:** 2026-07-05

**Authors:** Magdy Abdelmohsen Elsayed Mohamed, Mina Adolf Helmy, Omar Mohamed Elsayed Abou Hashem, Ahmed Mounir Shash, Emad Sedik Osman, Ahmed Ebrahim Mohamed, Mai Mohamed Ramzy Taha

**Affiliations:** 1 Department of Anesthesia and Critical Care Medicine, Cairo University, Cairo, Egypt; 2 Department of Anesthesia and Critical Care Medicine, Theodor Bilharz Research Institute, Cairo, Egypt

**Keywords:** Ambulatory surgery, Spinal anesthesia, Prilocaine, Bupivacaine, Fentanyl, Inguinal hernia, Urinary retention, Discharge readiness, Diseases, Health care, Medical research

## Abstract

Ambulatory inguinal hernia repair requires anesthetic techniques that provide reliable intraoperative analgesia while facilitating rapid recovery and timely discharge. Postoperative spontaneous voiding is a critical determinant of discharge readiness. This study compared unilateral intrathecal prilocaine–fentanyl with bupivacaine–fentanyl in male patients undergoing elective inguinal hernioplasty. In this randomized comparative trial, 70 male patients (ASA I–II, aged 18–60 years) scheduled for elective unilateral inguinal hernia repair were allocated to receive either intrathecal prilocaine 40 mg plus fentanyl 25 µg (Pr‑F group, n = 35) or bupivacaine 7.5 mg plus fentanyl 25 µg (Bu‑F group, n = 35). Blocks were performed unilaterally with patients maintained in the lateral decubitus position for 15 min. The primary outcome was time to first spontaneous voiding. Secondary outcomes included block characteristics, recovery parameters, pain scores, rescue analgesia, discharge readiness (Modified Post-Anesthesia Discharge Scoring System ≥ 9), and adverse events. Baseline demographics and intraoperative hemodynamics were comparable between groups. The Pr‑F group demonstrated significantly shorter time to first spontaneous voiding (213 ± 27 vs. 307 ± 22 min, p < 0.001). Motor and sensory regression times were also shorter with prilocaine (98 ± 11 vs. 187 ± 19 min; 120 ± 11 vs. 209 ± 23 min, respectively; p < 0.001). Time to achieve Modified Post-Anesthesia Discharge Scoring System ≥ 9 was reduced in the Pr‑F group (97 ± 11 vs. 187 ± 19 min, p < 0.001). Pain scores were higher with prilocaine, and rescue analgesia was required more frequently (34% vs. 11%, p = 0.023). Kaplan–Meier analysis confirmed earlier voiding in the Pr‑F group (log‑rank χ²=82.85, p < 0.0001). Adverse events, including pruritus, shivering, urinary retention, and transient neurological symptoms, were infrequent and comparable between groups. Unilateral intrathecal prilocaine–fentanyl provided faster recovery of sensory and motor function, earlier spontaneous voiding, and shorter discharge times compared with bupivacaine–fentanyl in male patients undergoing ambulatory inguinal hernia repair. Both regimens were safe, but prilocaine–fentanyl offers distinct advantages for fast‑track hernia surgery. Larger multicenter studies are warranted to confirm these findings across broader patient populations.

*Trial registration:* The study was registered retrospectively at ClinicalTrials.gov (Identifier: NCT07262398) on 14/11/2025.

## Introduction

Abdominal wall hernias remain a common surgical problem, with an overall prevalence of 1.7% across all ages and rising to 4% in individuals over 45 years. Inguinal hernias represent nearly 75% of all abdominal wall hernias^1^.

Spinal anesthesia has long been established as a reliable technique for inguinal hernia repair. It is simple to perform, provides a rapid onset, and ensures effective sensory and motor block in awake patients. With the growing emphasis on fast‑track, day‑case surgery, anesthetic techniques must not only provide adequate analgesia but also facilitate rapid recovery, early ambulation, and timely discharge^2^. This approach reduces healthcare costs, increases patient turnover, and enhances patient satisfaction. Consequently, the demand for short‑acting, potent anesthetic agents has become increasingly important.

Bupivacaine, an amide local anesthetic with intermediate onset and long duration of action, is widely used in spinal anesthesia for inguinal hernia repair. When administered in low doses (< 10 mg), intrathecal bupivacaine has been associated with shorter times to spontaneous voiding and discharge, although some patients still experience delayed recovery^3^. The addition of intrathecal fentanyl enhances analgesia through a synergistic effect, prolonging sensory block without significantly increasing sympathetic block or delaying recovery.

Prilocaine, another amide local anesthetic, offers a shorter duration of action and a favorable safety profile, with a low incidence of transient neurological symptoms^4^. These properties make it particularly suitable for fast‑track anesthesia. Furthermore, unilateral spinal block has been shown to accelerate recovery, reduce time to spontaneous voiding, and lower the incidence of temporary bladder catheterization compared with bilateral block^5^.

Given that inguinal hernias are far more prevalent in men, with a lifetime risk approaching one in four, male patients represent the majority of those undergoing surgical repair. Moreover, postoperative urinary retention, a key determinant of discharge readiness, is more frequently reported in men due to anatomical and physiological factors, making this population particularly relevant for evaluating anesthetic regimens that aim to optimize spontaneous voiding and safe discharge^6^.

Against this background, our study aims to compare unilateral intrathecal prilocaine–fentanyl with bupivacaine–fentanyl in male patients undergoing inguinal hernia repair within the ambulatory setting. The primary focus is on postoperative spontaneous voiding, a critical determinant of discharge readiness, assessed using the Modified Post Anesthesia Discharge Scoring System (MPADSS ≥ 9). By evaluating these regimens, we seek to identify the optimal anesthetic approach that balances efficacy, safety, and rapid recovery in fast‑track hernia surgery.

### Patient and methods

This randomized comparative study was conducted at Theodor Bilharz Research Institute and Cairo University Hospitals after obtaining approval from the TBRI Ethical Committee (PT 862). The study was registered retrospectively at ClinicalTrials.gov (Identifier: NCT07262398; registered on 14/11/2025). Written informed consent was obtained from each participant before enrollment.

All procedures involving human participants were performed in accordance with the ethical standards of the institutional and/or national research committee and with the 1964 Helsinki Declaration and its later amendments or comparable ethical standards. The study was designed and reported according to CONSORT guidelines.

Eligible participants were male patients aged 18–60 years, classified as ASA physical status I–II, with a body mass index below 35, scheduled for elective unilateral inguinal hernia repair using the standard open anterior prosthetic hernioplasty technique. Diagnosis was confirmed by ultrasonography. Patients were required to have no history of micturition disorders, and procedures were limited to less than 90 min. Exclusion criteria included allergy to study drugs, ASA III–IV status, bulky inguinal or inguinoscrotal hernias, infection at the injection site, non‑cooperative patients, pre‑existing neurological deficits in the lower extremities, abnormal coagulation profile, history of alcohol or substance abuse, contraindications or failure of spinal anesthesia, and incomplete data collection.

Seventy patients were randomized into two equal groups of thirty‑five each using computer‑generated numbers through an online randomization program. Allocation was concealed in opaque envelopes, which were opened only at the time of drug preparation by an anesthetist not involved in patient management. Intrathecal injections were performed under strict aseptic conditions.

Both patients and outcome assessors were blinded to group allocation. Importantly, the anesthesiologist who performed the spinal anesthesia was also blinded to the study drug, as preparation was conducted by a separate anesthetist. This ensured that drug administration, patient experience, and postoperative outcome assessment were all conducted under blinded conditions, thereby minimizing bias.

Group Pr‑F received prilocaine 40 mg (2 ml) combined with fentanyl 25 µg (0.5 ml) for a total volume of 2.5 ml, while Group Bu‑F received bupivacaine 7.5 mg (1.5 ml) combined with fentanyl 25 µg (0.5 ml) for a total volume of 2 ml. The spinal needle bevel was directed toward the dependent side to achieve a unilateral block, and patients remained in the lateral decubitus position for fifteen minutes before being repositioned supine.

Preoperatively, patients were asked to void their bladders. A 20‑gauge intravenous cannula was inserted, and 7 ml/kg lactated Ringer’s solution was infused. Baseline heart rate and blood pressure were recorded. Intraoperatively, standard monitoring, including ECG, pulse oximetry, and non‑invasive blood pressure, was applied. Oxygen was delivered via nasal prongs at 2 L/min, and Ringer’s lactate infusion continued at 4 ml/kg/hr. No sedation was administered. After local infiltration with lidocaine 2%, a 25-G Whitacre needle was inserted at L2–L3 or L3–L4 using a median approach, and free cerebrospinal fluid flow confirmed placement. Study drugs were injected slowly over sixty seconds.

Hypotension, defined as a decrease in systolic blood pressure by more than 20%, was treated with intravenous ephedrine 10 mg boluses. Bradycardia, defined as a heart rate below 45 beats per minute, was treated with atropine 0.01 g/kg, and such patients were excluded. Desaturation below 92% was recorded. Sensory block was assessed by pinprick testing, and motor block was evaluated using the Bromage scale. If the block was insufficient (< T10 after ten minutes), the operating table was tilted in Trendelenburg position; persistent failure led to conversion to general anesthesia and exclusion from the study.

At the end of surgery, patients received intravenous paracetamol 1000 mg for postoperative analgesia. In the post‑anesthesia care unit, vital signs and pain scores were recorded every twenty minutes. Shivering was treated with ondansetron 8 mg and dexamethasone 8 mg. Motor and sensory block regression was assessed every twenty minutes until full recovery, defined as a Bromage score of zero and sensory regression to the S2 dermatome. Patients were discharged once they achieved a Modified Post‑Anesthesia Discharge Score System (MPADSS) ≥ 9 and after spontaneous voiding. Retention requiring catheterization was documented.

Primary and secondary objectives:

Primary objective: The time to first spontaneous micturition, defined as the interval from completion of intrathecal drug injection to the patient’s first spontaneous voiding. Secondary objectives included peak sensory and motor block levels, need for table tilting, urinary catheterization, time to spontaneous voiding, time to achieve MPADSS ≥ 9, regression of sensory and motor block, surgeon satisfaction, time to first analgesic dose, transient neurological symptoms, incidence of shivering, pruritus, and postoperative nausea or vomiting.

### Sample size and statistical analysis

Sample size calculation was based on previous data showing a mean time to first micturition of 220 ± 47 min with prilocaine^7^. A sample size of sixty‑four patients (thirty‑two per group) was estimated to detect a 15% difference between groups with 80% power and α = 0.05. To account for dropouts, seventy patients (thirty‑five per group) were recruited.

Data were analyzed using SPSS version 23.0 (IBM Corp., Chicago, IL, USA). Quantitative variables were expressed as mean ± standard deviation for parametric data and median with interquartile range for non‑parametric data. Qualitative variables were expressed as numbers and percentages. Normality was tested using Kolmogorov–Smirnov and Shapiro–Wilk tests. Independent‑samples t‑test and Mann–Whitney U test were used for two‑group comparisons, paired t‑test for related samples, and Chi‑square or Fisher’s exact test for categorical variables. Time-to-event data for time to first void were analyzed using Kaplan–Meier survival curves. Differences between groups were assessed with the log-rank test. The chi-squared statistic and corresponding p-value were reported to evaluate statistical significance. A p‑value < 0.05 was considered statistically significant.

## Results

**Fig. 1:**
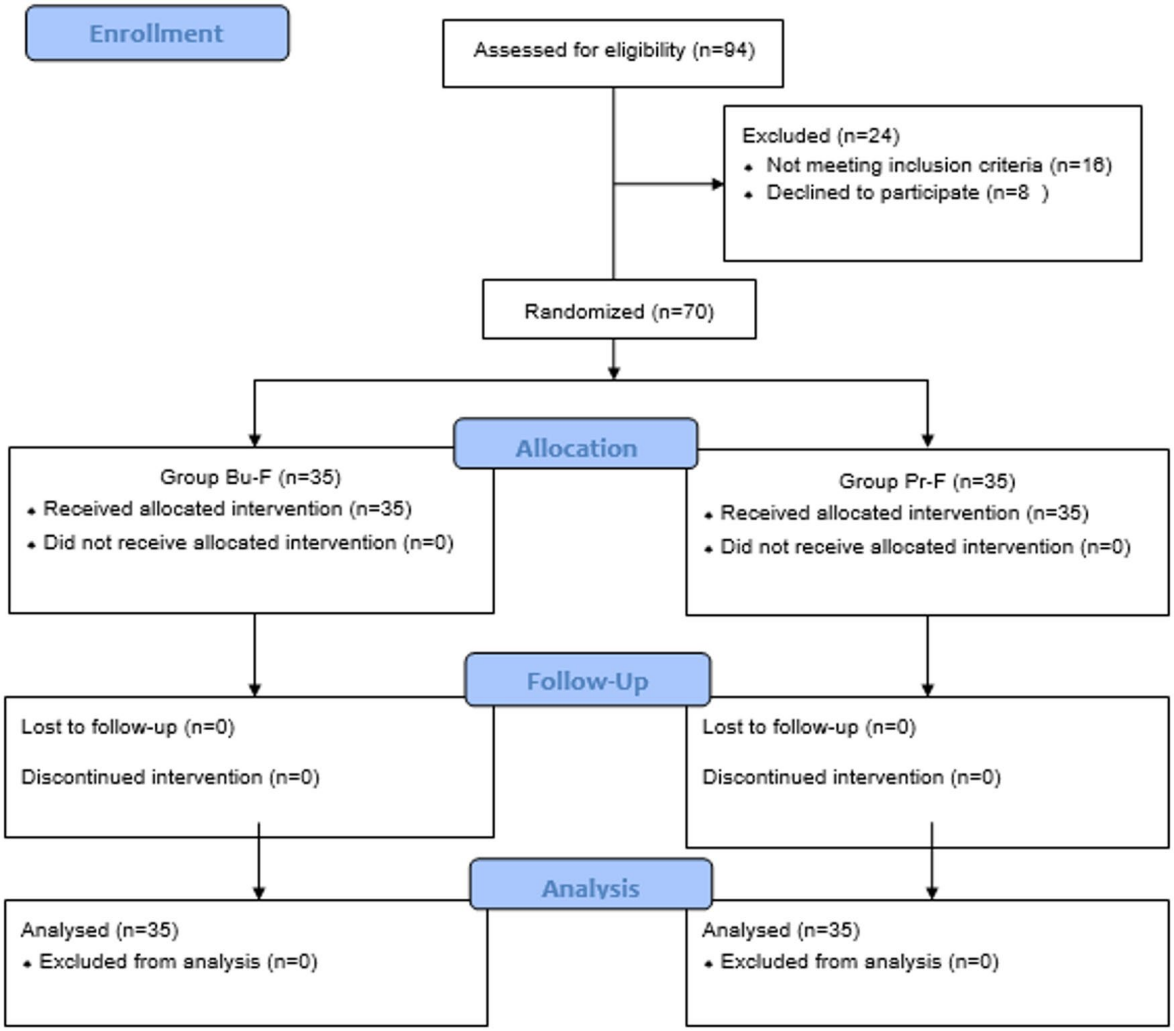
CONSORT flow chart of the included patients.

A total of ninety‑four male patients were screened for eligibility. Twenty‑four patients were excluded for not meeting the inclusion criteria, leaving seventy patients who were randomized equally into two groups: the Bupivacaine group (*n* = 35) and the Prilocaine group (*n* = 35) (Fig. 1).

**Fig. 2:**
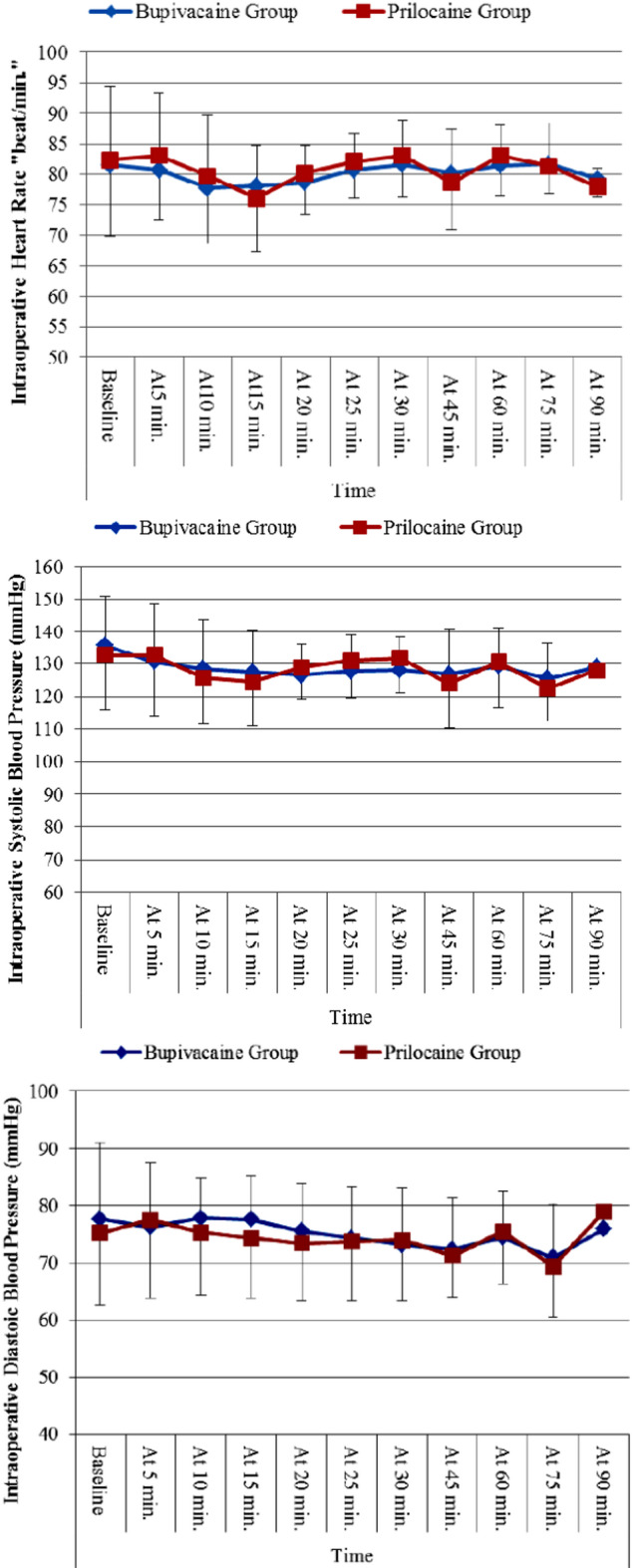
Intraoperative hemodynamics (heart rate, systolic blood pressure, and mean blood pressure).

Baseline demographic characteristics were comparable between the two groups, with no statistically significant differences in age, ASA physical status, weight, BMI, or duration of surgery (Table 1). Hemodynamic parameters, including systolic blood pressure, diastolic blood pressure, mean arterial pressure, and heart rate, remained stable throughout the perioperative period and showed no significant differences between groups (Fig. 2).

**Table 1:** Demographic data, data presented as mean ± SD and count (%).

Age (years)	Group Bu‑F (*n* = 35)	Group Pr‑F (*n* = 35)	*P* value
	45.09 ± 10.56	45.51 ± 8.46	0.852
ASA, n (%)			
I	31/35 (88.6%)	30/35 (85.7%)	0.721
II	4/35 (11.4%)	5/35 (14.3%)	
Weight (Kg)	83.29 ± 7.49	84.31 ± 9.47	0.616
BMI (Kg/m2)	27.97 ± 2.07	28.23 ± 2.57	0.647
Duration of surgery (min)	72.00 ± 11.39	71.14 ± 11.12	0.751

ASA: American Society of Anesthesiologists Physical Status Classification; BMI: Body mass index.

Assessment of sensory block revealed no statistically significant differences between the two groups in terms of dermatomal sensory level on the dependent side at 5 min, dependent side at 15 min, or non‑dependent side at 15 min (*p* > 0.05). However, within each group, the mean sensory level on the non‑dependent side at 15 min was significantly higher compared to the dependent side at 15 min (*p* = 0.001), indicating a consistent asymmetry in block distribution (Table 2). Motor block evaluation using the Bromage scale demonstrated no statistically significant differences between the two groups at 5–15 min on either the dependent or non‑dependent side (*p* > 0.05).

**Table 2:** Sensory and motor levels, data presented as mean ± SD and median[quartiles].

	Group Bu‑F (*n* = 35)	Group Pr‑F (*n* = 35)	*P* value
Sensory level			
DS at 5 min	6.69 ± 1.57	7.06 ± 1.45	0.308
DS at 15 min	6.11 ± 1.11	6.49 ± 1.04	0.152
NDS at 15 min	9.89 ± 1.32	9.89 ± 1.05	> 0.999
Bromage scale			
DS at 5 min	3[3–3]	3[3–3]	> 0.999
DS at 15 min	3[3–3]	3[3–3]	> 0.999
NDS at 15 min	2[1–2]	2[1–2]	0.188

DS: Dependent side; NDS: Non-dependent side.

Postoperative outcomes showed clear differences between the two groups (Table 3). Pain scores measured by the numeric rating scale (NRS) were consistently higher in the Prilocaine group at all time points from arrival to 120 min postoperatively (*p* = 0.001). The need for rescue analgesia was significantly greater in the Prilocaine group (34%) compared with the Bupivacaine group (11%, *p* = 0.023). Time to first analgesic request was markedly shorter in the Prilocaine group (92 ± 11 min) compared with the Bupivacaine group (165 ± 13 min, *p* = 0.001).

**Table 3:** Postoperative outcomes, data presented as mean ± SD, median[quartiles], and count (%).

	Group Bu‑F (*n* = 35)	Group Pr‑F (*n* = 35)	*P* value
NRS			
Arrival	0[0–0]	1[1–2]	0.001*
20 min	0[0–1]	2[1–2]	0.001*
40 min	1[0–1]	2[1–2]	0.001*
60 min	1[1–1]	2[1–2]	0.001*
80 min	1[1–1]	2[1–2]	0.001*
100 min	1[1–1]	2[1–2]	0.001*
120 min	1[1–1]	2[1–2]	0.001*
Need for rescue analgesia, n (%)	4/35(11%)	12/35(34%)	0.023*
Time to first analgesic request (min)	165 ± 13	92 ± 11	< 0.001*
Motor regression time (min)	187 ± 19	98 ± 11	< 0.001*
Sensory regression time (min)	209 ± 23	120 ± 11	< 0.001*
Time to first void (min)	307 ± 22	213 ± 27	< 0.001*
Time to MPADSS (min)	187 ± 19	97 ± 11	< 0.001*
Pruritus, n (%)	3/35(9%)	5/35(14%)	0.452
Post spinal shivering, n (%)	4/35(11)	2/35(6%)	0.393
Incidence of urinary retention, n (%)	0/35(0%)	0/35(0%)	> 0.999
Need for catheterization, n (%)	0/35(0%)	0/35(0%)	> 0.999
TNS, n (%)	0/35(0%)	0/35(0%)	> 0.999

NRS score – Numeric Rating Scale; MPADSS: Modified Post Anesthesia Discharge Scoring System; TNS: Transient neurologic syndrome.

*Denotes statistical significance.

**Fig. 3:**
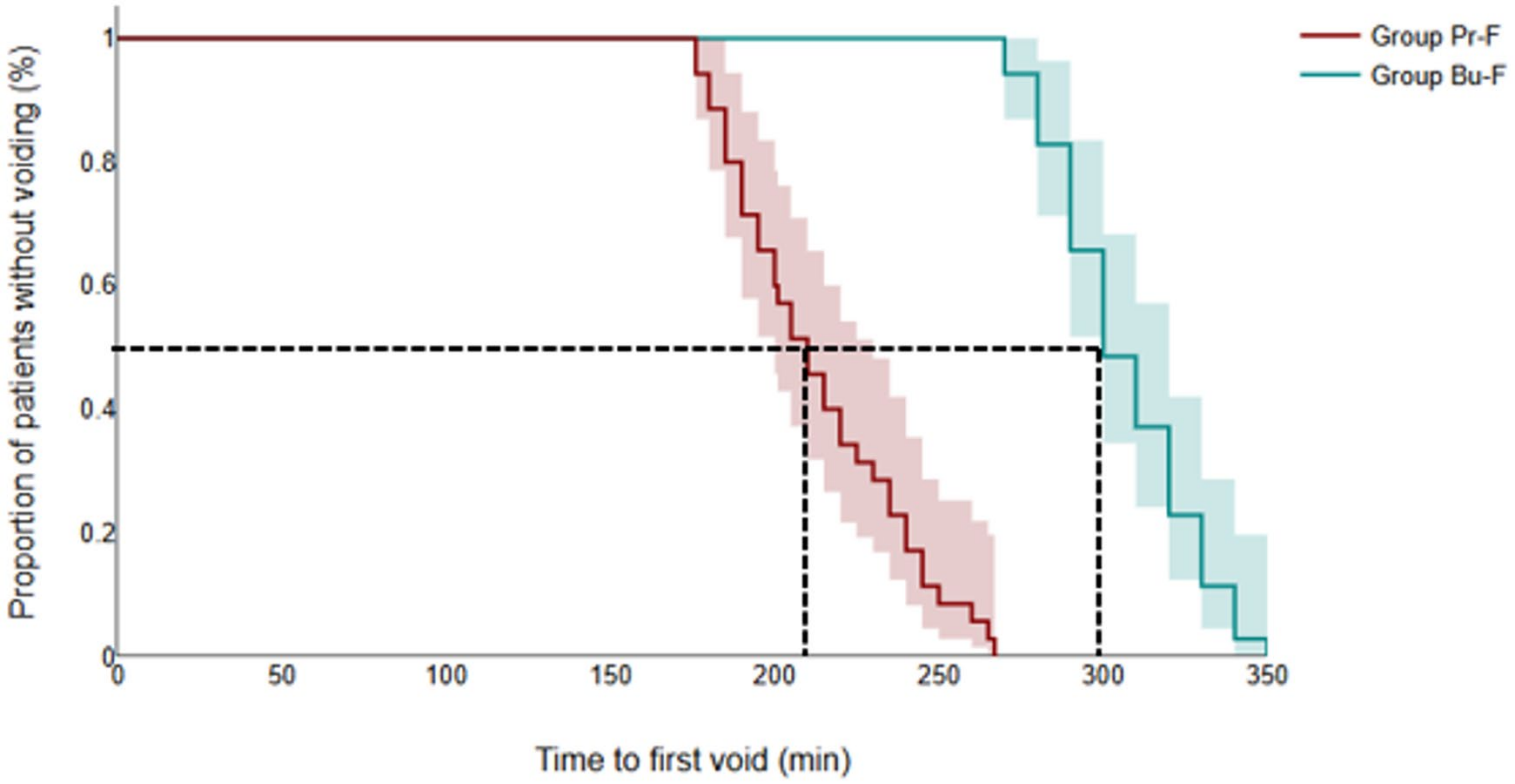
Kaplan–Meier curves for time to first void. The horizontal dashed line indicates the median recovery time, with the corresponding value shown at 50% recovery. Shaded areas indicated 95% confidence interval.

Kaplan–Meier survival analysis demonstrated a significant difference in time to first void between the two groups. The log-rank test yielded a chi-squared statistic of 82.85, indicating a significant difference (*p* < 0.0001) (Fig. 3).

Recovery parameters favored the Prilocaine group. Motor regression time was significantly shorter (98 ± 11 min vs. 187 ± 19 min, *p* = 0.001), as was sensory regression time (120 ± 11 min vs. 209 ± 23 min, *p* = 0.001). The primary outcome, time to first spontaneous voiding, was significantly reduced in the Prilocaine group (213 ± 27 min) compared with the Bupivacaine group (307 ± 22 min, *p* = 0.001). Similarly, time to achieve a Modified Post‑Anesthesia Discharge Score System (MPADSS) ≥ 9 was shorter in the Prilocaine group (97 ± 11 min) compared with the Bupivacaine group (187 ± 19 min, *p* = 0.001).

Adverse events were infrequent and comparable between groups. The incidence of pruritus, post‑spinal shivering, urinary retention, need for catheterization, and transient neurological symptoms did not differ significantly (*p* > 0.05).

## Discussion

Ambulatory surgery has become increasingly important in modern healthcare, driven by the need to reduce hospital stays, minimize costs, and improve patient satisfaction. In this context, anesthetic techniques must balance effective intraoperative analgesia with rapid recovery and minimal complications^8^. Spinal anesthesia remains a cornerstone for inguinal hernia repair, but the choice of local anesthetic significantly influences postoperative outcomes, particularly spontaneous voiding and discharge readiness.

In this randomized study, we compared unilateral intrathecal prilocaine–fentanyl with bupivacaine–fentanyl in male patients undergoing elective inguinal hernia repair. Baseline demographic and intraoperative parameters were comparable between groups, ensuring that differences in recovery profiles could be attributed to the anesthetic regimen.

The primary outcome, time to first spontaneous voiding, was significantly shorter in the prilocaine–fentanyl group (213 ± 27 min) compared with the bupivacaine–fentanyl group (307 ± 22 min, *p* < 0.001). This finding is clinically important, as delayed voiding is a common barrier to discharge in ambulatory surgery. Our results are consistent with Kotwani et al.^9^, who demonstrated faster voiding with low‑dose bupivacaine plus fentanyl compared to higher doses of bupivacaine alone. Similarly, Choi et al., in a systematic review, highlighted that neuraxial anesthesia transiently impairs bladder function, with recovery times correlating with anesthetic potency. Bupivacaine, being more potent and longer‑acting, was associated with prolonged detrusor blockade, whereas prilocaine’s shorter duration facilitated earlier bladder recovery^10^.

Interestingly, recent work showed that 40 mg hyperbaric prilocaine provided an earlier time to first void compared to 50 and 60 mg^11^.

Notably, no cases of urinary retention or catheterization occurred in either group, in contrast to reports of retention rates of 10–30% following spinal anesthesia. This may reflect standardized perioperative fluid management, avoidance of long‑acting opioids such as morphine, and vigilant bladder monitoring. The absence of retention underscores the safety of both regimens in the ambulatory setting.

Secondary outcomes further supported the superiority of prilocaine–fentanyl for fast‑track recovery. Sensory and motor block regression times were significantly shorter, leading to earlier ambulation and faster achievement of discharge criteria. These findings align with Kaban et al.^12^, who reported faster block regression and home readiness with prilocaine compared to bupivacaine in day‑case perianal surgeries. The unilateral technique likely contributed to these favorable outcomes by limiting cephalad spread and reducing the impact on sacral autonomic fibers, thereby accelerating bladder function recovery.

Pain scores were consistently higher in the prilocaine group, and rescue analgesia was required more frequently. However, this trade‑off was offset by the faster recovery profile and earlier discharge readiness. The addition of fentanyl in both groups enhanced sensory block quality without prolonging motor block or delaying recovery, ensuring adequate surgical anesthesia while preserving the advantages of a short‑acting local anesthetic.

Taken together, these findings support the use of prilocaine–fentanyl in unilateral spinal anesthesia for inguinal hernia repair in male patients. The regimen provided reliable anesthesia, faster recovery of bladder function, and earlier discharge compared with bupivacaine–fentanyl, without increasing adverse events. These advantages are particularly valuable in ambulatory surgery, where efficiency, patient comfort, and safety are paramount.

## Limitations

This study has limitations. First, the sample size was modest and limited to a single center, which may affect generalizability. Second, only male patients were included, reflecting the higher prevalence of inguinal hernias in men and their greater risk of postoperative urinary retention, but this restricts applicability to female patients. Third, the study focused exclusively on unilateral spinal anesthesia for inguinal hernia repair, and results may differ in other surgical procedures or with bilateral techniques. Fourth, follow‑up was confined to the immediate postoperative period, so long‑term outcomes such as persistent neurological symptoms or delayed urinary complications were not assessed. Fifth, although randomization and blinding of drug preparation, anesthesiologists, patients, and outcome assessors were rigorously applied, absolute blinding was not feasible. This limitation arises because the clinical characteristics of spinal blocks (such as onset time, intensity of motor block, and regression profile) may differ between prilocaine and bupivacaine, potentially allowing experienced anesthesiologists or patients to infer the allocated drug despite formal blinding procedures. Finally, while fentanyl supplementation allowed for reduced local anesthetic doses and enhanced intraoperative analgesia, it carries potential disadvantages such as pruritus, which may affect patient comfort and discharge readiness. In our study, pruritus was infrequent and did not differ significantly between groups. However, future trials evaluating prilocaine or bupivacaine without opioid adjuvants could provide further insight into optimizing anesthetic regimens for ambulatory hernia repair.

## Conclusion

Unilateral intrathecal prilocaine–fentanyl provided faster recovery of sensory and motor function, earlier spontaneous voiding, and shorter discharge times compared with bupivacaine–fentanyl in male patients undergoing ambulatory inguinal hernia repair. Both regimens were safe, with no cases of urinary retention or catheterization. Importantly, although pain scores were statistically lower in the bupivacaine group, both regimens maintained clinically well-tolerated pain levels (median NRS 0–2). These findings support prilocaine–fentanyl as a suitable anesthetic option for fast-track hernia surgery, offering advantages in patient comfort, efficiency, and healthcare resource utilization. Larger multicenter studies including both sexes and diverse surgical populations are warranted to confirm and extend these results.

## Data Availability

The datasets generated and/or analyzed during the current study are available from the corresponding author on reasonable request.
